# Application of matrix-assisted laser desorption/ionization time-of flight mass spectrometry in clinical testing and diagnosis

**DOI:** 10.3389/fcimb.2025.1607258

**Published:** 2025-11-10

**Authors:** Qiushuang Xiong, Haotong Guan

**Affiliations:** 1 Department of Clinical Laboratory, Union Hospital, Tongji Medical College, Huazhong University of Science and Technology, Wuhan, China; 2 Department of Gynecology, Zhongnan Hospital of Wuhan University, Wuhan, China

**Keywords:** MALDI-TOF MS, laboratory medicine, microbial identification, antimicrobial resistance, mutation detection

## Abstract

With the development of science and technology, new technologies are becoming more and more widely used in clinical testing. Matrix-assisted laser desorption ionization time-of-flight mass spectrometry (MALDI-TOF MS) is a technology with fast detection speed, low cost and high throughput. Since 2009, it has gradually entered the global clinical laboratory, and has gradually become an indispensable tool in clinical testing work, especially in clinical microbiology laboratory. In today’s field of microbial identification, MALDI-TOF MS fingerprinting analysis has become a fast and efficient means to identify microorganisms by comparing and analyzing the fingerprint of characteristic and stably expressed proteins with the constructed common reference spectrum library at the genus and species level. In addition to bacterial species identification, proteomic-based mass spectrometry also plays an important role in bacterial resistance detection by detecting antibiotic modification and hydrolysis of antibiotics and detecting changes in fingerprint maps of drug-resistant strains and sensitive strains. In terms of gene analysis, the platform of combined with PCR and MALDI-TOF MS can be used to identify viral infections and mutations, as well as clinical diagnosis of genetic diseases, including quantitative analysis of gene expression, analysis of gene copy number variation (CNV), and single-nucleotide polymorphism genotyping. Isothermal amplification-based MALDI-TOF MS is also expected as a complement to large-scale viral outbreak screening modalities. MALDI-TOF MS has played an enormous role in the identification of pathogenic microorganisms, virulence analysis, detection of bacterial resistance, study of resistance mechanisms, and clinical diagnosis of genetic diseases since its application, and it has been widely used in the diagnosis of bacterial diseases, it has brought revolutionary changes to the clinical diagnosis field and the rational application of antibiotics. The application of MALDI-TOF MS in clinical laboratory has significant advantages compared with traditional methods, but it also faces some challenges. In the future, with the continuous expansion and optimization of fingerprint database and the development and improvement of industry standards, MALDI-TOF MS will play a more important role in the field of clinical microbial testing and even clinical diagnosis.

## 1 Introduction

**Figure 1:**
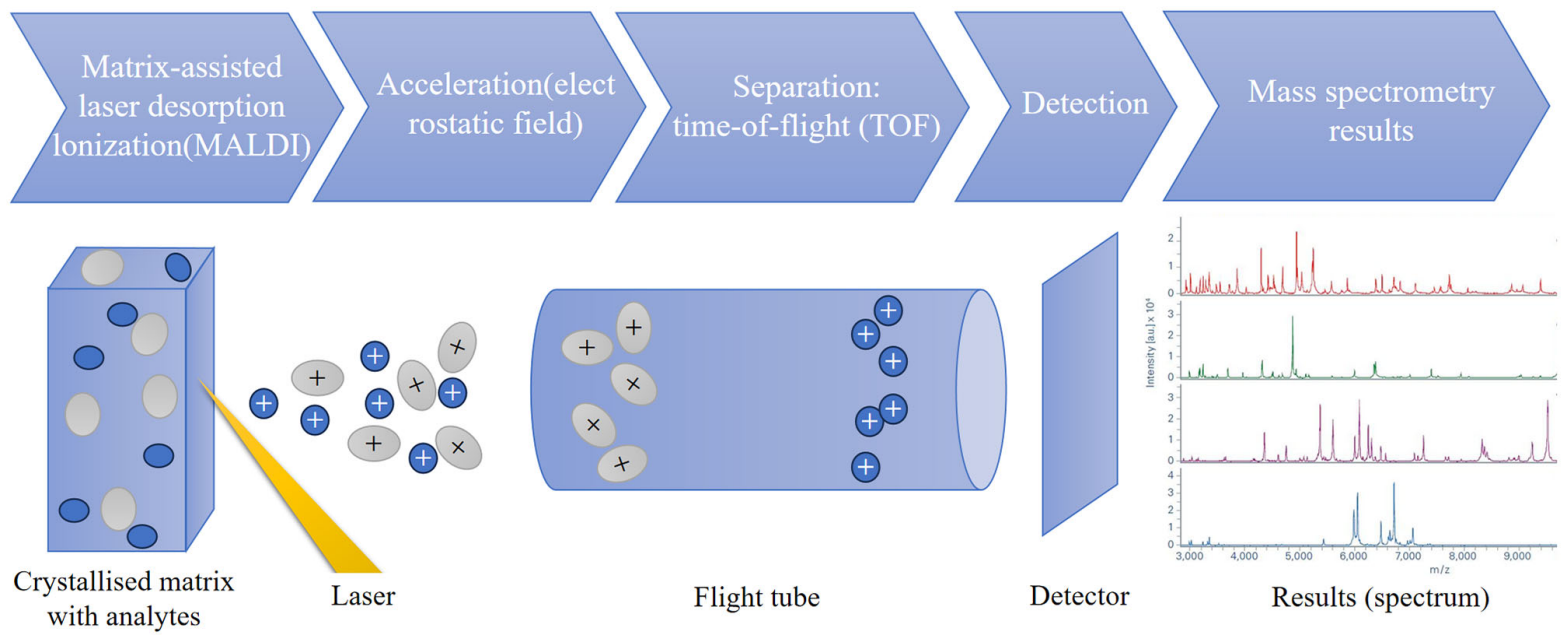
The basic principle of MALDI-TOF MS. MALDI-TOF MS crystallizes samples in an inert carrier matrix and uses a brief laser pulse to desorb and ionize characteristic ions (MALDI). The ionized molecules are accelerated in an electric field and separated in a vacuum tube. Their flight speed is proportional to the square root of the mass-to-charge ratio (m/z). The flight time (TOF) of ions is measured to determine their m/z. The electrical signals are processed by a computer and converted into a mass spectrum. The mass acquisition range is mainly from m/z 2000 to 20000. MALDI generates singly charged (z = 1) ions, so the m/z of the analyte corresponds to its mass value.

Since the late 1980s, matrix-assisted laser desorption/ionization (MALDI) time-of-flight (TOF) mass spectrometry (MS) has been applied in many scientific research fields, mainly in proteomics research. MALDI-TOF MS crystallizes the sample in an inert carrier matrix and then uses a short pulse laser of a specific wavelength to bombard the crystalline layer, causing desorption and soft ionization (MALDI). The soft ionization feature enables the complete ionization of molecules, which on the one hand maintains the integrity of the molecules and on the other hand avoids interference from fragments. The selection of the matrix and the matching of laser parameters are the basis for ensuring the ionization efficiency and detection sensitivity. The ionized molecules are accelerated in a high-voltage electric field andenter a vacuum flight tube. Their flight speed is proportional to the square root of the mass-to-charge ratio (m/z). The flight time (TOF) of ions is measured to determine their m/z. The electrical signals are processed by a computer and converted into a mass spectrum with m/z as the abscissa and ion flow peak intensity as the ordinate (Croxatto et al., 2012; Singhal et al., 2015)(**Figure 1**). Since Anhalt and Fenselau first used mass spectrometry for bacterial identification in 1975, the results were not reproducible due to the influence of culture conditions (Anhalt and Fenselau, 1975). It was not until 2009 that MALDI-TOF MS officially entered clinical laboratories (Bizzini and Greub, 2010; Croxatto et al., 2012). In 2013, MALDI-TOF MS was formally approved by the FDA for the identification of aerobic Gram-negative, some Gram-positive bacteria, and yeasts from cultures. Due to its superior accuracy in high-confidence identification of bacteria and greatly reduced sample turnaround time, MALDI-TOF MS has gradually begun to be widely used in clinical microbiology laboratories (van Belkum et al., 2015).With the continuous development and improvement of mass spectrometry technology, research on the use of MALDI-TOF MS for rapid identification of clinical bacteria, fungi, etc. has been increasing. In today’s microbiological research and clinical identification, MALDI-TOF fingerprint analysis has become a rapid and efficient method, especially suitable for the identification of ribosomal proteins in bacteria and fungi within the mass range of 2 to 20 kilodaltons (kDa) (Clark et al., 2013; Jhaveri et al., 2024). MALDI Biotyper (Bruker Daltonics) and VITEK MS (BioMerieux, Marcy-l’Etoile) based on the principle of MALDI-TOF MS are the most widely used bacterial identification systems in clinical laboratories (Calderaro and Chezzi, 2024). By analyzing the unique “fingerprint” signature protein profiles formed by highly abundant and stably expressed proteins such as ribosomal proteins in microorganisms (e.g., bacteria), compared with the universal reference spectral library constructed in the database, the rapid and reliable identification of microbial species can be realized. The cost of MALDI-TOF MS equipment is comparable to that of other diagnostic laboratory equipment (such as RT-PCR equipment), but the cost per sample is lower. Compared with conventional biochemical identification that takes up to 72 hours to produce results, the time for bacterial species identification using MALDI-TOF MS is reduced from several days to a few minutes, and results are usually obtained within 24 hours after sample collection (Croxatto et al., 2012; Sauget et al., 2017), significantly shortening the time of conventional biochemical identification and bringing a revolutionary breakthrough to clinical diagnosis (Osthoff et al., 2017). However, the speed of MS is not at the expense of accuracy. While ensuring the identification speed, it is reliable for the identification of common and rare clinical bacterial and fungal groups, and also greatly reduces the detection cost. Therefore, it plays an increasingly important role in clinical microbiological examination.

### 1.1 Application in microbial strain identification

The traditional microbial strain identification was mainly based on morphology(such as cell morphology, staining reaction), ecological characteristics (such as source environment, growth conditions and pathogenicity) and biochemistry (such as metabolic pathway, enzyme activity), which had the defects of tedious operation and long detection cycle. Therefore, significant efforts have been devoted to the development of rapid, accurate and high-throughput technologies for the identification of pathogenic microorganisms. MALDI-TOF MS fulfills the requirements for fast detection, high sensitivity and high accuracy, and has become one of the standard methods for microbial species identification. Existing standard methods are only applicable to single colony analysis after purification, multibacterial samples must be purely cultured for typical MALDI-TOF MS identification, and species identification in mixed microbial communities is still in the experimental stage (Yang et al., 2018; Papagiannopoulou et al., 2020). For the identification of pure culture strains using MALDI-TOF MS, pretreatment is usually required to inactivate pathogenic microorganisms, destroy their cell walls, and extract intracellular proteins. Due to the different structure and composition of the cell wall of different types of microorganisms, the difficulty of breaking the cell wall is different, which directly affects the release of proteins. Therefore, an appropriate pre-treatment solution is needed for the pretreatment process, among which the formic acid-acetonitrile extraction method has a wide application range (Rudrik et al., 2017). The main functions of the pretreatment solution were to inactivate pathogenic microorganisms, destroy cell wall, release intracellular proteins, reduce impurity interference, and optimize mass spectrometry quality. In order to obtain high-quality mass spectra, suitable matrix is needed after optimizing protein extraction. A suitable matrix molecule must be able to co-crystallize with the analyte and, crucially, possess a strong molar absorptivity at the wavelength of the laser used. Traditional matrices such as a-cyano-4-hydroxycinnamic acid(CHCA), sinapinic acid (SA) and 2,5-dihydroxybenzoic acid (DHB) exhibit significant selectivity for analytes of different molecular weights, and the pretreatment process needs to be adjusted according to the type of microorganism.

In addition to rapid identification of pure cultures, MALDI-TOF MS can directly detect microorganisms in clinical samples including positive blood culture specimens, urine specimens, cerebrospinal fluid, pleural and peritoneal fluid, and other body fluids, without the need for culturing, and with only simple sample prep steps such as centrifugation and lysis of hemocytes, which greatly shortens the detection time (Prod'Hom et al., 2010; Osthoff et al., 2017; Idelevich et al., 2018; Mörtelmaier et al., 2019; Lamy et al., 2020). Although the types and quantities of bacteria in the sample, sample pretreatment methods, and whether it is a mixed infection will affect the detection rate, the direct identification of positive blood culture specimens based on pure colonies is relatively mature, which can shorten the turnaround time for rapid identification and meet the needs of rapid clinical diagnosis and treatment. While a limited number of studies have reported that MALDI-TOF MS can be used to identify various microorganisms in polymicrobial blood cultures, the accuracy of identification may be significantly compromised when the number of bacterial species exceeds two or when the microbial proportions are highly imbalanced. A study published in 2019 evaluated the performance of *Bruker*^®^*MBT Sepsityper IVD* for the direct identification of pathogens in mixed microbial blood cultures using MALDI-TOF MS. The results showed that 49 (34.3%) of 143 mixed blood culture samples were simultaneously identified with two species, and more than 50% of the samples were identified with at least one species (Scohy et al., 2018).The pretreatment method was improved by incorporating short-term enrichment in liquid culture medium, followed by MALDI-TOF mass spectrometry for the identification of bacteria and yeasts in polymicrobial blood cultures. In samples containing two microorganisms, 86% (43/50) achieved accurate identification of both species, thereby improving the overall identification accuracy (Florio et al., 2019). Another study proposed a novel framework based on linear regression for analyzing MALDI-TOF spectra of bacterial mixtures without requiring a purification step. Statistical evaluation using Jackknife resampling demonstrated that 95% of species were correctly identified in double mixtures with balanced cell counts, and 86% in triple mixtures under similar conditions. In mixtures with asymmetric cell distributions, the dominant species was consistently identified (Yang et al., 2018). The Bruker^®^ Sepsityper system enables reliable identification of two-species mixtures. However, identification accuracy declines when more than two bacterial species are present or when the abundance ratio is highly skewed, such as in cases where one species is overwhelmingly dominant. The identification of slow-growing bacteria, such as mycobacteria, has been one of the difficulties in clinical microbiology due to the time-consuming isolation culture and routine biochemical identification. Whereas MALDI-TOF MS enables the rapid and accurate identification of mycobacteria, including pathogenic mycobacterial complex (MTC) and non-tuberculous mycobacteria (NTM), as well as the identification of the majority of microbiota, the ability to obtain identification results in only a few hours of sample preparation time after colony formation improves the level of mycobacterial identification (Alcaide et al., 2018; Gonzalo et al., 2023).

MALDI-TOF MS has also become a primary tool for the rapid and accurate identification of a wide range of clinically relevant yeasts (e.g., *Can-dida* spp., *Cryptococcus* spp.) and is increasingly used for filamentous fungi (e.g., *Aspergillus* spp., *Fusarium* spp.). while identification of yeasts is often straightforward, filamentous fungi can require more rigorous protein extraction protocols due to their robust cell walls. However, the technology has proven superior to traditional morphological and biochemical methods in terms of speed and accuracy for many fungal species (Bader et al., 2011; Bader, 2013; Cassagne et al., 2016). With the development of MALDI-TOF MS technology, the optimization of algorithms, and the update of database versions, significant progress has been made in the identification of common clinical fungi. Mark Fraser et al. used the MALDI Biotyper platform to identify 6328 pathogenic yeast isolates with 93.6% accuracy (Fraser et al., 2016). After direct extraction with formic acid, all 309 clinical isolates of *Candida* spp. and uncommon yeasts were identified at a comparable rate using two commonly used platforms, Biotyper and VITEK MS; however, for the identification of uncommon yeast isolates, the identification rate was improved with an improved extraction method, acetonitrile formic acid extraction (Lee et al., 2017). The identification of clinical fungi has been improved, in particular, the ability of MALDI-TOF MS to distinguish between closely related species within certain genera (such as *Candida, Cryptococcus*, *Fusarium*, and *Aspergillus*) is increasing and can be used as a preferred method of identification (Bizzini and Greub, 2010; De Carolis et al., 2012a; Gautier et al., 2014; Burckhardt and Zimmermann, 2018; Patel, 2019). Since the prognosis of patients in the clinic largely depends on the rapid and accurate diagnosis of the condition, and MALDI-TOF MS just meets this need, it has shown great application potential in the field of clinical microbial identification.

Although MALDI-TOF MS is still in the research phase for parasitic infections and not yet a routine clinical tool, it has shown growing potential in identifying specific parasites (e.g., *Leishmania*, *Giardia*, *Cryptosporidium*, amoeba) and arthropod vectors (Cassagne et al., 2014; Sánchez-Juanes et al., 2022; Torrico et al., 2023). It can distinguish *Plasmodium*-infected from uninfected *Anopheles* mosquitoes, identify nematodes like *Trichinella* and *Dirofilaria*, and detect intestinal parasites such as *Giardia* via protein profiling (Laroche et al., 2017; Feucherolles et al., 2019). However, most published studies are exploratory or proof-of-concept and lack large-scale clinical validation against gold standard methods. While MALDI-TOF MS shows promise for parasite identification, challenges remain: certain parasites, particularly helminths, exhibit complex life cycles with significantly varying protein expression profiles across developmental stages. Moreover, for some species, obtaining sufficient quantities of pure samples is difficult due to limitations in *in vitro* cultivation or the labor-intensive process of isolating and purifying parasite eggs or larvae from clinical specimens such as feces. To date, standardized and widely accepted pretreatment protocols for different parasite species or sample types have not been established, which greatly limits result comparability and impedes broader technological adoption. Consequently, the clinical implementation of MALDI-TOF MS for parasite identification remains challenging and requires further refinement before widespread use can be realized.

## 2 Detection of antibiotic resistance

For the treatment of pathogenic bacteria, the early selection of appropriate antibiotics is crucial for the prognosis of infection. The research methods of MALDI-TOF MS for antibiotic resistance detection mainly include two aspects: detecting the modification and hydrolysis of antibiotics to identify the bacteria producing drug-resistant enzymes, and detecting the fingerprint changes of resistant and sensitive strains (De Carolis et al., 2012a; Florio et al., 2018a). In recent years, there have been many reports in this field, including the differentiation of methicillin-resistant *Staphylococcus* aureus(MRSA)and methicillin-sensitive *Staphylococcus* aureus(MSSA), the differentiation of vancomycin-resistant *Enterococcus*(VRE) and vancomycin-susceptible *Enterococcus* (VSE), the detection of β-lactamases in Gram-negative bacilli, the detection of outer membrane proteins in *Enterobacteriaceae*, and the detection of ribosomal protein mutations in *Escherichia* coli (Hrabák et al., 2011; Camoez et al., 2016; Tang et al., 2019; Candela et al., 2022; Del Corpo et al., 2023). In the detection of drug-resistant bacteria, the test strain is co-incubated with β-lactam antibiotics, and the hydrolysis of the antibiotics is determined based on the change in molecular weight to evaluate the activity of β-lactamases. By detecting the hydrolysis of antibiotics, it can predict the presence or absence of a specific resistance mechanism (Oviaño and Bou, 2019). Different bacteria can form different protein profiles by MALDI-TOF MS, and whether they are resistant can also affect the specific peaks of the strain. Some bacterial species have biomarkers related to resistance, such as MRSA, Klebsiella pneumoniae producing KPC, and Bacteroides fragilis with positive *cfiA* gene. By detecting characteristic peaks, the resistance or sensitivity of a certain antibiotic can be quickly predicted (Yoon and Jeong, 2021). By directly detecting the structural modifications of lipid A molecules related to specific resistance mechanisms (especially resistance to polymyxin antibiotics), it also enables rapid prediction of the resistant phenotype. In addition, recent studies have shown that rapid antibiotic susceptibility testing (AST) can be performed by comparing the MALDI-TOF MS spectra of pathogenic bacteria with and without co-incubation with antibiotics (Edmondson et al., 2024; Zhang et al., 2025). Under specific incubation conditions or in combination with other technologies (such as nucleic acid-based detection or machine learning-assisted analysis), this method is applicable to the detection of antibiotic resistance for a variety of antibiotics and related pathogens (Cao et al., 2018). The MALDI Biotyper rapid antibiotic susceptibility test is a semi-quantitative rapid AST test. By exploring the appropriate concentration and time of antibiotic incubation and calculating the area under the curve (AUC) of the spectra after incubation with or without antibiotics by software, the growth of microorganisms can be evaluated (Oviaño and Bou, 2019). If the microorganism is sensitive, due to growth inhibition, the AUC of the spectrum after incubation with antibiotics will be lower than that of the control without antibiotics; for resistant strains, the effect of antibiotics is small, and the AUC of the spectra is similar regardless of whether they are co-incubated with antibiotics. Through software prediction, the time for determining antibiotic resistance can be significantly shortened (Hao and Ying, 2024). Based on the accumulation of MALDI-TOF MS data, machine learning can perform more complex analysis and better distinction of spectra through modeling for resistance prediction.

Over the past decade, while more attention has been focused on the identification of bacteria and fungi, as well as the detection of bacterial drug resistance, MALDI-TOF MS technology has also made some progress in the detection of antifungal drug resistance. Evolving from an initial tool for microbial identification, it has gradually become a multifunctional platform with potential for both antibacterial and antifungal resistance detection. Around 2015, the application of MALDI-TOF MS in antifungal drug resistance detection was still in the exploratory stage. Early research mainly concentrated on proof-of-concept and method development, with researchers attempting to assess fungal drug resistance by comparing the changes in their mass spectra before and after drug treatment (De Carolis et al., 2012b; Florio et al., 2018a, 2018). Although MALDI-TOF MS holds potential for detecting antimicrobial resistance, studies during this period have been less advanced for fungi compared to bacteria (Singhal et al., 2015; Florio et al., 2018b). From 2018 to 2020, researchers increasingly focused on the standardization and optimization of MALDI-TOF MS for antifungal resistance detection. During this period, progress was made in refining key parameters such as sample pretreatment, drug exposure duration, and concentration selection. Furthermore, novel algorithms were developed for mass spectral comparison and resistance classification, reducing manual intervention while enhancing detection efficiency and reproducibility (Florio et al., 2018b; Li et al., 2022).From 2020 to 2023, MALDI-TOF MS for antifungal resistance detection entered the clinical validation stage. During this phase, multiple clinical laboratories participated in evaluating the accuracy and reliability of resistance detection using MALDI-TOF MS, and dedicated studies were conducted on antifungal resistance in clinically important fungi such as *Candida and Aspergillus* (Florio et al., 2018b; Knoll et al., 2021). Subsequently, MALDI-TOF MS technology began transitioning toward commercial application, exemplified by the development and implementation of commercial kits such as MBT ASTRA (MALDI Biotyper Antibiotic Susceptibility Test Rapid Assay) (Vatanshenassan et al., 2019). Although technical challenges remain in areas including database coverage, sample processing, identification of mixed microorganisms, and detection of specific resistance mechanisms, these limitations are being progressively addressed through expanded and optimized databases, advances in novel technologies and methodologies, the rollout of commercial solutions, and integration with complementary analytical platforms.

## 3 Applied to the diagnosis and quantification of viral infections

For the identification and quantification of viral infection, it is difficult to distinguish and identify by conventional MALDI-TOF MS because of the narrow molecular weight range of viral capsid protein and the small difference in protein profiles between different viral strains. During the outbreak of COVID-19, differentially expressed proteins enriched for neutrophil degranulation and acute inflammatory response functions in COVID-19 patients, and that differential protein detection of the features could enable rapid identification of infection during a pandemic (Shen et al., 2020). Another study was conducted to analyze serum peptides from COVID-19 patients and controls using MALDI-TOF MS. The accuracy, specificity, and sensitivity of the machine learning model constructed based on 25 differential characteristic peaks to identify COVID-19 patients in independent tests were greater than 95%, and the detection cost per sample was less than $1, with only 5 μL of serum required to complete the analysis in less than 1 minute (Yan et al., 2021). The MALDI- TOF MS-based serum peptide analysis in this study eliminated the need for an additional clean testing environment, had a low risk of sample cross-contamination, and reduced the risk of exposure to the sampler.

Furthermore, regarding virus detection, the low viral load during the early stages of infection—such as a limited number of viral particles in blood or secretions—presents a challenge. Traditional MALDI-TOF MS may struggle to directly detect low-abundance viral gene fragments due to its sensitivity being dependent on the concentration of the target molecule within the sample. Adriana et al. showed that viral enrichment in cell culture and ultracentrifugation to concentrate viral particles for the detection of respiratory viruses was effective in differentiating between uninfected and respiratory virus-infected cell cultures, including parainfluenza, influenza, and adenoviruses (Calderaro et al., 2016). In contrast, nucleic acid detection methods like PCR can enhance sensitivity by amplifying the nucleic acid fragment. Due to the high specificity and sensitivity of PCR and the high throughput, high accuracy, and flexibility of MALDI-TOF MS, PCR-MALDI-TOF MS has been developed as an alternative method for the detection of SARS-CoV-2 (Wang et al., 2020; Hernandez et al., 2021). The findings of Rybicka et al. showed that the PCR-MALDI-TOF MS-based assay had higher specificity in SARS-CoV-2 detection compared to RT-qPCR, which was widely used during the global outbreak, and it has the advantage of label-free and simultaneous detection of SARS-COV-2 multimutations (Rybicka et al., 2021). PCR-coupled MALDI-TOF MS: The workflow of this technique begins by extracting viral DNA or RNA from clinical samples. For RNA viruses, it is necessary to first convert them into more stable complementary DNA (cDNA) through reverse transcription, and then use PCR to amplify the target-specific regions. A key feature of this amplification is that it is usually performed in the presence of dideoxynucleotide trisphosphate (dNTPs) and dideoxynucleotide trisphosphate (ddNTPs) in a process similar to Sanger sequencing. After extension, a series of specific small DNA fragments are generated, whose quality is determined by the template sequence. After purifying the extension products and removing the interference of salt ions, they are analyzed by MALDI-TOF MS. The precise quality of each fragment reveals the underlying nucleotide sequence at a specific site, enabling detection of the virus and identification of a specific genotype or mutation. Further optimization on the basis of the widely used “Gold standard” real-time fluorescent quantitative PCR (qPCR) technique, Guobin Han’s group proposed a new method for SARS-COV-2 detection by isothermal amplification in combination with MALDI-TOF MS, under conditions that do not require cyclic temperature changes, has been achieved, to achieve a rapid and high-specificity detection of target genes l (Han et al., 2022). Ultimately, the target molecule or the amplified product of the target molecule can be detected directly, and in addition, no probe or fluorescent dye labeling is required, largely avoiding the false-positive results generated by the background signa. While this approach provides greater flexibility and speed compared to traditional methods, its ability to identify new isolates relies on existing reference spectra in databases.

For viral drug resistance detection, localized and quantitative allele frequency assays using MALDI-TOF MS in hepatitis B virus (HBV)-infected populations to study the genotypic evolution of pre-existing HBV quasispecies and drug-resistant variants during treatment with nucleoside(t) analogs were monitored with sensitivity up to 100 copies/mL, which can help to further the understanding of the disease progression and the response to the drugs (Hong et al., 2004; Rybicka et al., 2014, 2015). In addition, the assessment of the treatment status of HIV-infected individuals through virus genotyping analysis based on MALDI-TOF MS has a lower detection limit than traditional sequencing methods. It is more sensitive and accurate for monitoring the emergence of HIV genotypic variations that cause drug resistance and for early judgment (Lee et al., 2013). The detection of nucleic acid sequence mutations by MALDI-TOF MS requires converting different alleles into nucleic acid fragments with distinct mass differences. After specifically amplifying the DNA fragments containing the target mutation site through techniques such as PCR, there are two strategies. One is through single base extension reaction (SBE), where the final molecular weight of the extension product will depend on the base type at that site of the template strand. The other is to use base-specific enzymes or chemical reagents to cut the PCR products. The presence of different allele sequences will lead to different cleavage patterns or fragment lengths of the cleavage products, thereby generating a mixture of fragments with different molecular weights, which can be finally distinguished by MALDI-TOF MS. Nucleic acid mass spectrometry, as a molecular diagnostic technique, can work with the same instrument as microbial mass spectrometry but needs to be coupled with specialized reagent consumables (Hao and Ying, 2024). Overall, MALDI-TOF MS, with its advantages of high throughput and low cost, has broad application prospects in the rapid diagnosis of infectious diseases and drug resistance monitoring. However, the construction of standardized databases and clinical validation in the field of virology still need to be strengthened.

## 4 Diagnosis of genetic diseases and tumors

In terms of genetic analysis, the platform based on PCR and MALDI-TOF MS (Agena Bioscience) has been applied in various genetic and epigenetic studies as well as clinical diagnosis of genetic diseases, these include quantitative analysis of gene expression, copy number variation (CNV) analysis, single nucleotide polymorphism (SNP) genotyping, and DNA methylation identification (Roscioli et al., 2013; Clendenen et al., 2015; Zuo et al., 2015; Yang et al., 2021; Udomsinprasert et al., 2023). MALDI-TOF MS is used in genetic analysis for genetic screening of inherited deafness in newborns, genetic testing for nasopharyngeal cancer risk, and molecular diagnosis of Familial adenomatous polyposis(FAP),those which by detecting specific gene mutation sites, it provides a basis for the early discovery and prevention of diseases (Bonk et al., 2002; Tang et al., 2010; Yao et al., 2014; Huang et al., 2016; Tian et al., 2023). Additionally, in tumor research and treatment, MALDI-TOF MS is used to detect mutations or methylation status of tumor-related genes, and to select appropriate targeted drugs for precise medication in tumor diagnosis and treatment (Largy et al., 2022; Liu et al., 2024). In-source decay (ISD) occurs in the ion source during the instant of laser desorption/ionization in matrix-assisted laser desorption ionization (MALDI). It represents a novel and distinct fragmentation technique that differs from conventional methods such as collision-induced dissociation (CID) or post-source decay (PSD). Combined with the ISD fragmentation technology, the complete protein can be directly fragmented in the ion source, and the sequence analysis of the complete protein can be carried out directly without enzymatic hydrolysis, it preserves the complete information of the Posttranslational modification and can effectively locate and identify the Posttranslational modification, which are closely related to the occurrence and development of many diseases (Théberge et al., 2015; John et al., 2020). Theoretically, MALDI-TOF MS combined with in-source decay has great potential in discovering and identifying protein isoforms and modified forms that are directly related to diseases. Nine biomarker proteins associated with persistent urothelial bladder cancer, including Apo a 1, were screened by Nedjadi et al. using two-dimensional differential gel electrophoresis (2D-DIGE) combined with MALDI-TOF MS (Nedjadi et al., 2021). Zaki et al. established a plasma peptidomic map of breast cancer and identified three peptides (m/Z 1570.31,1897.40, and 1568.17) from the model to distinguish breast cancer patients from healthy controls with 96.4% accuracy (Zaki et al., 2019). Timms et al. found that combined detection of the two characteristic peaks of ovarian cancer MALDI-MS and the serum routine marker CA125 predicted ovarian cancer 11–15 months earlier than CA125 alone (Bonk et al., 2002). After enrichment of prostate cancer samples and controls with hydrophilic interaction chromatography nanoparticles (HICNPs), Sun et al. detected a significant difference in the MALDI-TOF signal, with an analytical accuracy of 77%, approaches based on prostate-specific antigen (Sun et al., 2020). A statistical model by Forajta et al. showed that a classification accuracy of 83.3% to 100.0% could be achieved for patients with prostate cancer and healthy population by using preselected MS signals (Buszewska-Forajta et al., 2022). Statistically significant differences in total serum protein fingerprinting based on MALDI-TOF MS were found between hepatocellular carcinoma patients and healthy controls (Park et al., 2019). Due to the high throughput and low cost of the MALDI-TOF MS assay, it will have a promising application in large-scale tumor screening. The application of MALDI-TOF MS in clinical laboratory as presented in **Table 1**.

**Table 1:** Summary of Clinical Applications of MALDI-TOF MS.

Type of detection	The application in clinical laboratory	Comment	Reference
Bacteria and fungi	Identification	Positive blood culture samples, urine samples, cerebrospinal fluid, pleural fluid and ascites samples were directly detected.	Prod'Hom et al., 2010; Osthoff et al., 2017; Idelevich et al., 2018; Mörtelmaier et al., 2019; Lamy et al., 2020
		Other clinical specimens were cultured and purified for detection.	Yang et al., 2018; Papagiannopoulou et al., 2020
	Antimicrobial resistance testing	The modification of antibiotics and the molecular weight changes of hydrolysates were detected.	De Carolis et al., 2012b; Florio et al., 2018a; Oviaño and Bou, 2019
		Characteristic peaks of biomarkers related to drug resistance were identified.	Yoon and Jeong, 2021
		The area under the curve (AUC) of the spectra before and after the co-incubation of antibiotics was compared to quickly evaluate the drug susceptibility.	Oviaño and Bou, 2019
Viruses	Diagnosis and mutation detection of viral infections	Host response characteristic proteins or viral gene fragments were detected by MALDI-TOF MS after virus culture or *in vitro* gene amplification.	Yan et al., 2021; Shen et al., 2020; Calderaro et al., 2016; Wang et al., 2020; Hernandez et al., 2021; Rybicka et al., 2021
		The detection of HBV and HIV gene mutations after drug treatment can reach a sensitivity of 100 copies/mL.	Rybicka et al., 2015; Lee et al., 2013
Genetic diseases	Detection of genetic mutations and genotyping	Genetic screening for hereditary deafness in newborns, genetic testing for nasopharyngeal cancer risk, molecular diagnosis of familial adenomatous polyposis	Bonk et al., 2002; Yao et al., 2014; Huang et al., 2016; Tian et al., 2023
Tumors	Classification of tumor patients and healthy populations by characteristic difference peaks	Earlier prediction of ovarian cancer by combined CA125 and MALDI-MS detection of two characteristic peaks.	Bonk et al., 2002
		Classification of prostate cancer patients and healthy populations after enrichment of samples with HICNPs.	Sun et al., 2020
		Nine biomarkers associated with persistent urothelial bladder cancer were identified	Nedjadi et al., 2021
		The established breast cancer plasma peptide map could distinguish breast cancer patients with 96.4% accuracy	Zaki et al., 2019
		Classification of hepatocellular carcinoma patients and healthy populations by total serum protein fingerprinting	Park et al., 2019

## 5 Limitations in application to clinical testing

Common methods for pathogen microorganism identification include morphology, biochemistry, MALDI-TOF MS and gene sequencing. Currently, fully automatic microbial biochemical identification instruments and MALDI-TOF MS are widely used in laboratories. While established DNA sequencing methods are routine and increasingly affordable for comprehensive genomic analysis, MALDI-TOF MS offers a highly efficient, cost-effective, and high-throughput alternative for targeted applications. Its strengths lie in the rapid analysis of specific genetic variations, such as single-nucleotide polymorphism (SNP) genotyping, copy number variation (CNV) analysis, and the quantitative assessment of DNA methylation status, which are critical for genetic disease diagnosis and pharmacogenomics. At present, the accuracy of MALDI-TOF MS identification is close to that of molecular identification methods including sequencing, and higher than biochemical identification. Despite the relatively high instrument cost, MALDI-TOF MS offers lower overall expenses for bacterial and fungal testing compared to conventional methods, along with advantages in broader coverage of pathogenic microorganisms, faster detection speed, higher identification accuracy, lower detection cost and higher detection throughput.

Although MALDI-TOF MS technology has brought revolutionary changes to clinical testing, it still has some limitations (**Table 2**). Firstly, the principle of MALDI-TOF MS technology is based on the unique peaks formed by the relative molecular mass of the proteins of the tested strain. Therefore, when the relative molecular mass of the proteins of two species is similar(such as Escherichia coli/Shigella, Streptococcus pneumoniae/Streptococcus mitis), MS may have difficulty in accurate identification and may cause confusion and misjudgment, requiring supplementary tests or serological tests for comprehensive judgment (Devanga Ragupathi et al., 2018; Sadowy and Hryniewicz, 2020). Secondly, the quality of the obtained spectra also depends on the choice of matrix, the composition of the matrix solvent and the sample preparation procedure. The matrix solvent can promote the release of macromolecules within the cells, while the co-crystallization of the matrix compound and the target molecule determines the mass range of the ionized molecules, thereby enhancing the differential ionization effect of specific compounds such as proteins, peptides or nucleotides (Chac et al., 2021; Han et al., 2021). Traditional matrices have obvious selectivity for analytes with different molecular weights, and the suitable matrix is the prerequisite for obtaining high-quality mass spectra. For example, CHCA is generally regarded as the preferred matrix for analyzing low-molecular-weight peptide segments (< 3000 Da) and small proteins, which can provide extremely high ionization efficiency and detection sensitivity for low-abundance peptide segments; SA can better protect large molecules from fragmentation, making it an ideal choice for analyzing high-molecular-weight proteins (> 2500 Da, often up to 100 kDa or above); DHB has a wide range of applications and is commonly used for analyzing glycoproteins, glycopeptides, phosphorylated peptides, and some medium-molecular-weight proteins. One of its significant advantages is its high tolerance to sample contaminants such as salts and detergents. In addition, mucus colonies may dilute the bacterial content on the target plate due to the presence of their capsules, resulting in unsatisfactory identification results. It may be necessary to selectively pick samples for coating or cleaning, centrifugation, and enrichment prior to identification. This makes it impossible to unify the pretreatment methods of different strains that differ in structure. Thirdly, a key challenge lies in the requirement for microbial isolation and cultivation.Although the identification of colonies after purification can be achieved, it is still difficult for the identification of multiple mixed bacteria, and mixed samples are difficult to analyze accurately (Yang et al., 2018; Mörtelmaier et al., 2019; Reeve and Bachmann, 2019). In mixed samples, culture-based purification is required to improve spectral quality, reproducibility, and identification accuracy. Furthermore, the identification accuracy is contingent upon the inclusion of the corresponding fingerprint in the database, and the identification of certain rare microorganisms largely relies on the comprehensiveness and timeliness of database updates. These indicate that researchers in clinical microbiology laboratories should have a full understanding of the limitations of MALDI-TOF MS and, if necessary, establish a localized database and select appropriate pretreatment methods based on the characteristics of the microorganisms to be identified.

**Table 2:** Limitations of MALDI-TOF MS in clinical laboratory application.

Application types	Mainly limitations	Potential solutions
Bacteria and fungi	MALDITOF MS was difficult to identify accurately when the relative molecular mass of proteins from the two strains was similar.	Complementing other identification methods such as biochemical identification, serological detection.
	Pure strains are required, mixed strains have an effect on the identification results.	post-culture purification
	The differences in the structure of different strains make the pretreatment methods different and can not be unified.	Selection of the appropriate pretreatment is based on the initial judgment of staining and morphological examination.
	Depend on the integrity and update degree of the database.	Update database regularly and establish localized database.
Viruses	The accuracy of the identification depends on whether the corresponding fingerprint is included in the database.	Update database regularly and establish localized database.
	Low viral load limits detection in the early stage of viral infection.	cultured and enriched for detection.
Genetic diseases and tumors	Highly random variants could not be comprehensively detected.	MALDI-TOF MS was employed for the initial screening of known gene loci, and subsequently, challenging cases were validated using sequencing technologies, such as whole-genome sequencing (WGS) or whole-exome sequencing (WES).
	Relying on databases that are analyzed against them.	Extending and optimizing the database; optimizing the selection of feature peaks for comparison by machine learning.

## 6 Discussion

With the rapid development of medical science and technology, clinical laboratory automation has become an important trend in modern medical testing. Matrix-assisted laser desorption ionization time-of-flight mass spectrometry (MALDI-TOF MS) technology plays an increasingly important role in clinical laboratory automation with its unique advantages. Combining high sensitivity, high resolution and fast analysis, this technology enables efficient sample analysis and data processing in automated processes for clinical laboratories, thus significantly improving laboratory efficiency and accuracy. In the automated system of clinical laboratories, MALDI-TOF MS technology is mainly used for microbial identification, drug resistance analysis, and genetic analysis. Through the automated steps of sample pretreatment, spotting, mass spectrometry analysis, and data interpretation, it realizes the full-process automation from sample receipt to report issuance. This not only greatly shortens the detection time but also reduces human errors, improving the reliability and reproducibility of the detection.

MALDI-TOF MS in the detection of clinical microorganisms, mainly based on proteomics mass spectrometry analysis. With the advantages of simplicity, rapidity, low cost and reproducibility, MALDI-TOF MS plays a great role in strain identification, drug resistance monitoring and identification of mutant strains. The main advantage of MALDI-TOF MS is that compared with biochemical identification-based methods, it greatly shortens the identification time while guaranteeing the identification accuracy, and it has great advantages in the identification of anaerobic and fastidious microorganisms. In situations where the strain grows slowly, traditional methods are difficult to identify, and in life-threatening infections, it can save more precious time for the diagnosis of patients’ conditions and the selection of medications. With the development of MALDI-TOF MS technology, optimization of algorithms, and version updates of databases, significant progress has been made in the identification of common clinical fungi, especially among closely related species within certain genera (such as Candida, Cryptococcus, Fusarium, and Aspergillus), with an increasing ability to differentiate them, which can be used as the preferred method of identification. However, the accuracy of identification is also largely dependent on the database of the comparison, and the continuous improvement of the database, as well as the laboratory’s self-constructed library can effectively improve the accuracy and reliability of the detection.

MALDI-TOF MS applied to nucleic acid detection has the advantages of high throughput and multi-indicator detection, and can complete the detection of a large number of samples in a short period of time. At the same time, this technology does not require fluorescent or radioactive labeling, which reduces the experimental cost and operational complexity, and can share the platform with microbial mass spectrometry, which only requires replacement of consumables. Therefore, nucleic acid detection based on isothermal amplification of MALDI-TOF MS is expected to be applied in large-scale epidemiological studies of different viruses as an effective complement to the existing methods, and has a good potential to be applied in the clinical diagnosis of interspecies gene mutation and genetic diseases. In addition, it has good application prospects in interspecies gene mutation and clinical diagnosis of genetic diseases. However, there are also some challenges in nucleic acid mass spectrometry, in addition to the need to continuously improve and update the reference database in order to improve the accuracy and reliability of the test. In terms of technology standardization, it is also necessary to develop uniform operation specifications and quality control standards to ensure the comparability of results between different laboratories.

Future progress and wider adoption of MALDI-TOF MS in the clinical setting will be driven by continued, multifaceted development across several domains. This includes the engineering of more sensitive and higher-resolution mass spectrometers (hardware development); the creation of more robust, standardized, and automated sample processing protocols and workflows (assay development); and the crucial expansion and curation of comprehensive spectral databases along with more sophisticated analytical algorithms (software and database development). These parallel advancements are necessary to overcome current limitations and unlock the full diagnostic potential of the technology. With the continuous development and improvement of mass spectrometry technology, MALDI-TOF MS will play an even more important role in clinical pathogenic organisms detection, providing powerful support for rapid diagnosis and precise treatment of diseases. In the clinical microbiology laboratory, the limitations of this technology should be fully understood, localized databases should be established if necessary, and appropriate pretreatment methods should be selected according to the characteristics of the target strain. The application of MALDI-TOF MS in clinical laboratory needs to be optimized and explored, and it also has great potential in the field of virus detection in the future.
