# Supplementary material for: Application of matrix-assisted laser desorption/ionization time-of flight mass spectrometry in clinical testing and diagnosis
Source: Front Cell Infect Microbiol. 2025 Nov 10;15:1607258. doi: 10.3389/fcimb.2025.1607258 (PMC12640996; doi:10.3389/fcimb.2025.1607258)

**Supplementary Table**

**TABLE.S1. Summary of the performance of MALDI-TOF MS applied to virus-related detection in the manuscript.**


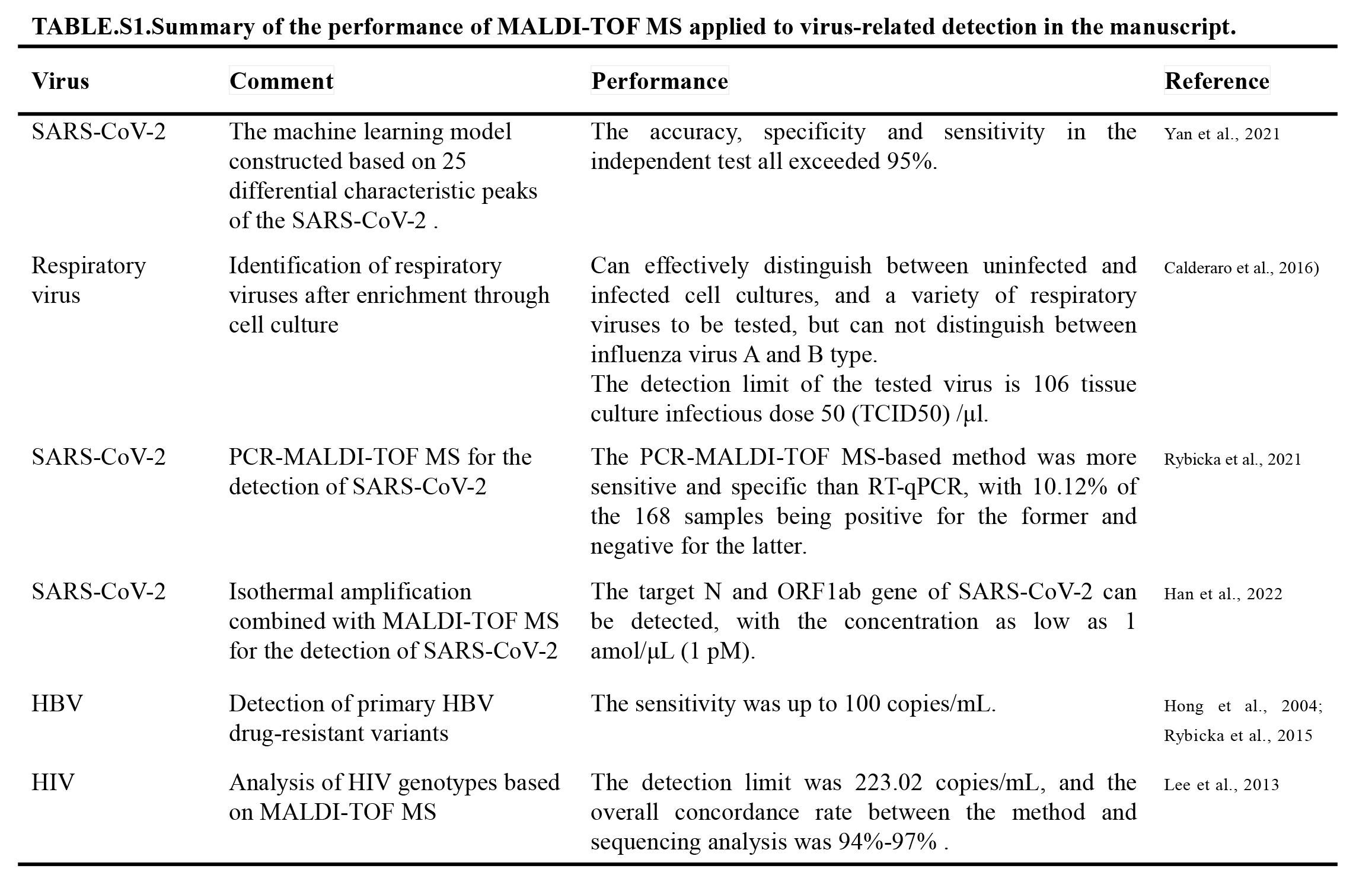

Supplement: Supplementary file 1 [file Table1.docx]
